# Investigating the Effect of Substituting a Single Cysteine Residue on the Thermal Stability of an Engineered Sweet Protein, Single-Chain Monellin

**DOI:** 10.1007/s10930-023-10154-0

**Published:** 2023-09-22

**Authors:** Kyosuke Ohnuma, Atsuko Yamashita, Norihisa Yasui

**Affiliations:** 1 School of Pharmaceutical Sciences, Okayama University, Okayama, Japan; 2 Graduate School of Medicine, Dentistry and Pharmaceutical Sciences, Okayama University, 1-1-1, Tsushima-naka, Kita-ku, Okayama, 700-8530 Japan

**Keywords:** Crystallography, Monellin, Protein Stability, Recombinant Proteins

## Abstract

Single-chain monellin (SCM) is an engineered protein that links the two chains of monellin, a naturally sweet-tasting protein. This protein is an attractive candidate for use as a sugar replacement in food and beverages and has numerous other applications. Therefore, generating SCM mutants with improved stability is an active area of research to broaden the range of its potential applications. In this study, we focused on the Cys41 residue of SCM, which is a single cysteine residue present at a structurally important position. This residue is often substituted with Ser. However, this substitution may destabilize SCM because Cys41 is buried in the hydrophobic core of the protein. Therefore, we designed mutants that substituted Ala, Val, and Leu for this residue, namely C41A, C41V, and C41L. We characterized these three mutants, SCM C41S, and wild type (WT). Differential scanning fluorimetric analysis revealed that substituting Cys41 with Ala or Val increased the thermal stability of SCM, while substitution with Ser or Leu decreased its stability. Determination of the crystal structures of SCM C41A and C41V mutants revealed that the overall structures and main chain structures around the 41st residue of both mutants were almost identical to the WT. On the other hand, the orientations of the amino acid side chains near the 41st residue differed among the SCM variants. Taken together, our results indicate that substituting Cys41 with Ala or Val increases the stability of SCM and provide insight into the structural basis of this improvement.

**Supplementary Information:**

The online version contains supplementary material available at 10.1007/s10930-023-10154-0.

**Abbreviations**: RMSD, root mean square deviation; SCM, single-chain monellin; SDS-PAGE: sodium dodecyl sulfate polyacrylamide gel electrophoresis.

## Introduction

**Fig. 1:**
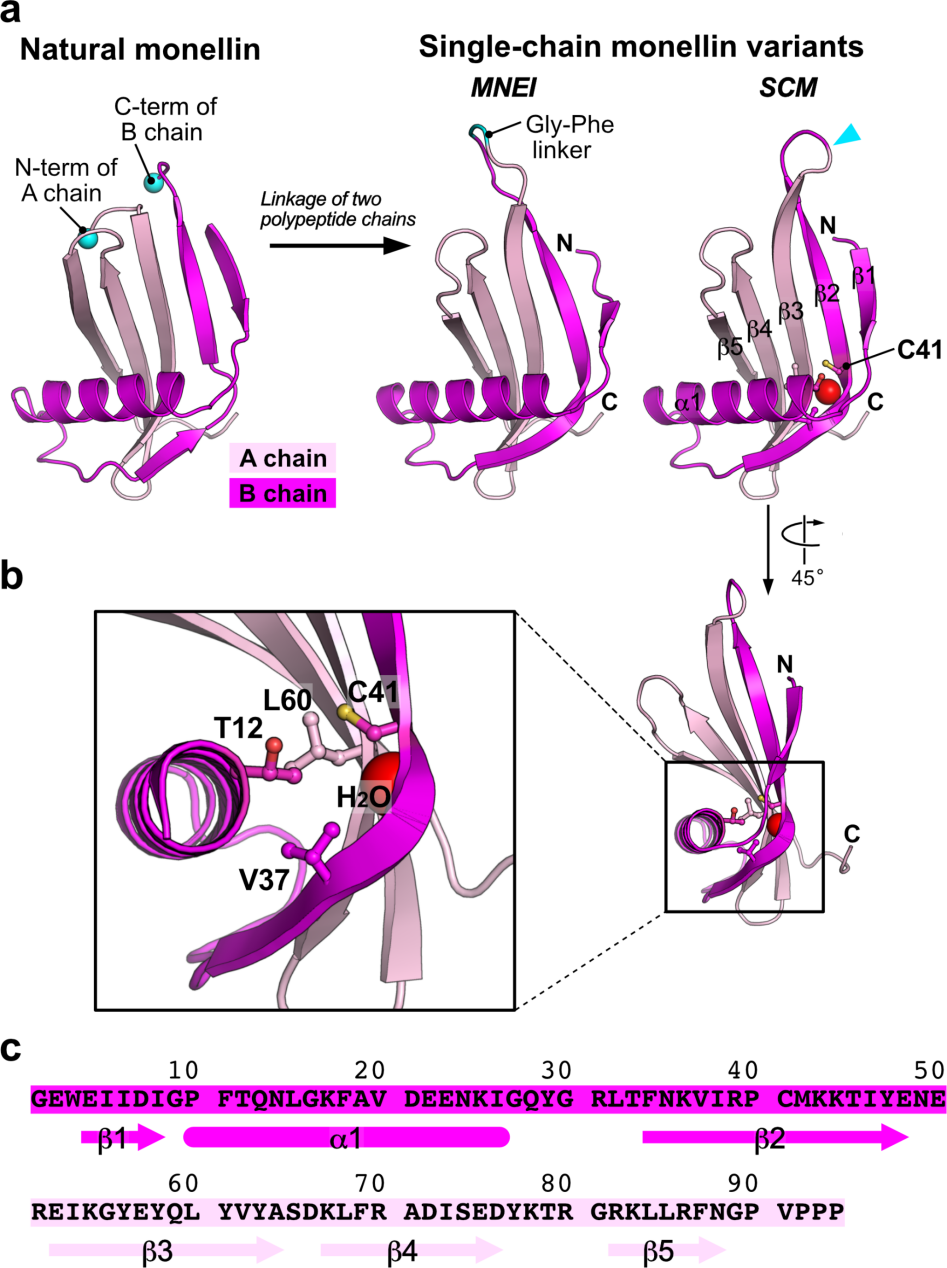
**Design of SCM variants. a** Overall structures of natural monellin (*left*, PDB ID: 3MON), MNEI (*middle*, PDB ID: 2O9U), and SCM (*right*, PDB ID: 1MOL). The portions derived from the A and B chains are colored light pink and magenta, respectively. The C-terminus of the B chain and the N-terminus of the A chain in the structure of natural monellin are shown as cyan spheres and labeled. Both polypeptide chains are connected by a loop between the β2 and β3 strands to generate single-chain variants of monellin, MNEI and SCM. The Gly-Phe linker segment in MNEI is colored cyan and labeled. A cyan arrowhead indicates the position of the link in the SCM structure. **b** Close-up view of Cys41 residues. The side chains of the amino acid residues around the Cys41 residue are indicated using ball and stick models. The oxygen atom of the water buried in the protein, assigned as W309 in the 1MOL coordinate, is presented as a red sphere. Residues Ile6 – Gly9 were omitted from the coordinate to illustrate the figure for clarity. **c** Amino acid sequence of the wild type of SCM. The secondary structural elements are indicated below the amino acid sequence

Monellin is a naturally sweet-tasting protein isolated from the fruit of *Dioscoreophyllum cumminsii*, a plant indigenous to West Africa [1]. This sweet protein comprises two noncovalently associated polypeptide chains, namely the A chain, and B chain, which comprise 44, and 50 amino acid residues, respectively (UniProt IDs: P02881 and P02882 for the A chain and the B chain, respectively). Structural analyses using X-ray crystallography have revealed that the N-terminus of the A chain and the C-terminus of the B chain are positionally close to each other [2] (Fig. 1a, *left*). Consequently, the engineered protein called single-chain monellin (SCM) has been generated in which the C-terminus of the B chain is directly linked to the N-terminus of the A chain (Fig. 1a, *right*, and c) [3]. The crystal structure revealed that the fold of SCM is similar to that of the natural monellin, as designed [4] (Fig. 1a, *right*). Another type of single-chain variant of monellin, MNEI, has also been engineered; in this molecule, the A and B chains are linked via a Gly-Phe linker instead of the direct linkage used in SCM [5] (Fig. 1a, *middle*). The number of residues differs between the two single-chain variants, 94 residues for SCM and 96 residues for MNEI, depending on the presence of a Gly-Phe linker sequence connecting the two polypeptide chains. The solution structure and high-resolution crystal structure of MNEI revealed that this variant also has a fold similar to those of natural monellin and SCM [6, 7] (Fig. 1a).

Single-chain variants of monellin have been used for various applications due to their unique molecular properties. It has been reported that these variants elicit a strong sweet taste and are more stable than natural monellin in terms of changes in temperature and pH [3]. Therefore, these proteins have gained attention for use as sugar substitutes in foods and beverages; however, the range of possible applications of single-chain variants of monellin remains limited due to its solubility and aggregation properties [8]. In addition to its use as a sugar substitute, one of the single-chain variants of monellin, SCM, can also be used to generate antibody-like binder proteins that act as a molecular scaffold in which the amino acid sequence and the length within the two loops are randomized [9]. In such an application, it is desirable to stabilize SCM-based scaffolds since increasing the lengths of loops with diversified amino acid sequences tends to decrease binder stability [9]. In addition, single-chain variants of monellin have also been used as model proteins for protein folding, unfolding, and aggregation studies [10–12]. This is because they have two types of secondary structures, α-helix and β-sheet, and the effect of fusing two separate polypeptide chains into a single chain can be studied [13].

Mutagenesis has been employed to improve the thermal stability of single-chain variants of monellin, especially for the purpose of sugar replacement in foods and beverages. Indeed, numerous combinatorial mutations have been reported in single-chain variants of monellin. These mutants maintain the sweet taste but increase the thermal stability of the protein [14–16]. The Cys41 residue is especially noteworthy among potential mutation sites. First, in native monellin, Cys41 is located on the β2 strand of chain B, where it contacts the β3 strand of chain A (Fig. 1a, *right*). Second, there is Pro40 residue just before Cys41 (Fig. 1c), which results in a kink in the β2 strand at this position [4]. Third, there is a completely buried water molecule between the β2 and β3 strands [4] that forms hydrogen bonds with the main-chain atoms of Ile38 (O), Pro40 (O), and Tyr63 (N) and can also interact with the S atom of Cys41 [7] (Fig. 1b). Fourth, Cys41 is the only cysteine residue present in natural monellin or its single-chain variants (Fig. 1c).

Heterologous expression of single-chain variants of monellin performed in host cells (e.g., in *Escherichia coli*) may result in intermolecular disulfide bond formation mediated by Cys41, leading to undesirable aggregation. To overcome this issue, Ser or Ala residues have been substituted for Cys41 [9–11, 17–21]. Nevertheless, substitution with a polar residue like Ser may destabilize the molecule since the side chain of the 41st residue is completely buried in the core of the protein molecule, forming part of the interface between the β-sheet and α-helix [4]. In fact, the polar side chain of Ser at the 41st position is buried, as revealed by the crystal structures of the mutants of single-chain variants of monellin containing the C41S mutation [9, 14]. A viable alternative is to substitute the 41st residue with a hydrophobic residue. However, the effect on the thermal stability of substituting Cys41 with hydrophobic amino acids other than alanine has not yet been extensively studied.

In this study, we designed SCM mutants by substituting the Cys41 residue with three hydrophobic amino acid residues—i.e., Ala, Val, and Leu—generating SCM C41A, C41V, and C41L, respectively. Next, we purified these three mutant proteins along with WT SCM and C41S mutant prepared by recombinant overexpression in *Escherichia coli*. To test their dispersity in solution, we analyzed purified samples using size-exclusion chromatography. Subsequently, we estimated their melting temperatures and compared the thermal stability of SCM variants by differential scanning fluorimetry. Finally, we determined the crystal structures of the SCM C41A and C41V mutants, and compared the structures of these SCM mutants so that we could use their three-dimensional structures to investigate the effects of the substitution of Cys41 on thermal stability.

## Materials and methods

### DNA Manipulations

Expression vectors for SCM C41A, C41V, C41L, and wild type SCM (i.e., C41) were constructed based on pHFT-SCM C41S, which encodes an SCM whose 41st residue is Ser that also has a decahistidine, a FLAG tag, and a TEV cleavage site fused to its N-terminus [9]. Next, we introduced point mutations in SCM at the 41st residue by PCR, which was performed using KOD One (TOYOBO) and the primer sets shown in Table S1. All constructs were verified by DNA sequencing.

### Expression and Purification of SCM Mutants

SCM variants were expressed and purified according to a previously described method [9]. In brief, BL21(DE3) cells were transformed with expression vectors, and protein expression was induced using autoinduction media [22] for 22–24 h at 30 °C. His-tagged SCM variants were then purified from the soluble fraction using Ni-NTA agarose (QIAGEN). The tagged form of the SCM variants was subjected to TEV protease treatment, followed by application to a Ni-NTA agarose column, where the unbound fraction was collected.

### Size-Exclusion Chromatographic Analysis

The purified SCM or its mutants (at a concentration of 40 µM in 100 µL) were subjected to size-exclusion chromatography on an ENrich SEC 70 10 × 300 column (Bio-Rad) equilibrated with 20 mM HEPES-Tris, 150 mM NaCl, pH 7.5, at a flow rate of 1 mL/min with NGC Quest 10 Plus (Bio-Rad).

### Differential Scanning Fluorimetry

The thermal stability of SCM mutants was evaluated by differential scanning fluorimetry, as previously described [9], using the Protein Thermal Shift kit (Applied Biosystems). Briefly, the purified protein samples were dialyzed against the appropriate buffers. Specifically, the following buffers were used for each pH condition: 20 mM MES-Na, 150 mM NaCl, pH 5.0 (for acidic pH), 20 mM HEPES-Na, 150 mM NaCl, pH 7.5 (for near-neutral pH), and 20 mM Bicine-Na, 150 mM NaCl, pH 8.6 (for basic pH). The dialyzed protein (5 µg) and Protein Thermal Shift Dye were mixed in the dialysis buffer to prepare 20 µL of a protein melt reaction. A StepOne Real-Time PCR System (Applied Biosystems) was used to measure fluorescence intensity. The mixtures were denatured by raising the temperature from 25 to 99 °C at a rate of 0.022 °C/s. The apparent thermal denaturation temperatures (*T*_m_) were estimated using the two-state Boltzmann model as implemented in Protein Thermal Shift Software version 1.3 (Applied Biosystems).

### Protein Crystallization

Crystallization of the SCM C41A and C41V mutants was performed using the sitting drop vapor diffusion method, following the reported crystallographic analysis of SCM WT [4]. Briefly, the SCM C41A mutant protein was concentrated to 20 mg/mL in 20 mM Tris-HCl, pH 8.0, 300 mM NaCl, and then crystallized using a solution containing 0.1 M sodium citrate, pH 5.0, and 35% (w/v) PEG3350 at 20˚C. The SCM C41V mutant was at a concentration of 21 mg/mL in 20 mM HEPES-Na, pH 7.5, 150 mM NaCl, and was crystallized using a solution containing 0.1 M Bis-Tris, pH 6.0, 35% (w/v) PEG3350 at 20˚C.

### X-ray Crystallography

Crystals were cryoprotected by soaking them in mother liquor supplemented with 20% (v/v) ethylene glycol and then flash frozen in liquid nitrogen. X-ray diffraction data were collected at the SPring-8 (Harima, Japan) beamline of BL41XU at a wavelength of 1.0000 Å using an EIGER X 16 M (DECTRIS) detector using the ZOO automatic data collection system [23]. Diffraction data sets were processed with KAMO [24] for automatic data processing using XDS [25]. Initial phases were determined via molecular replacement with Phaser [26] in the CCP4 program suite using chain A from the coordinates of SCM WT (PDB ID: 1MOL) as the search model. Both crystals contained two molecules of each SCM variant in an asymmetric unit. The models were manually rebuilt with Coot [27] and refined with Phenix.refine [28]. A summary of the data collection and refinement statistics is shown in Table 1.

**Table 1:** Data collection and refinement statistics in X-ray crystallographic analysis

	SCM C41A (PDB 8JZ0)	SCM C41V (PDB 8JZ1)
Data collection*		
Space group	*P*1	*P*2_1_
Unit cell		
*a*, *b*, *c* (Å)	31.7, 39.7, 44.4	39.1, 48.7, 47.2
*a, β*, *γ* (˚)	106.2, 109.3, 103.4	90.0, 102.7, 90.0
No. of molecules/a.s.u	2	2
X-ray source	SPring-8 BL41XU	SPring-8 BL41XU
Wavelength (Å)	1.0	1.0
Resolution (Å)	50.0–1.23 (1.30–1.23)	50.00–1.24 (1.31–1.24)
Total reflections	117,644	277,760
Unique reflections	79,961 (5243)	81,703 (6581)
Completeness (%)	74.5 (30.3)	84.6 (42.1)
Redundancy	1.5 (1.4)	3.4 (2.9)
*R*_merge_ (%)	4.1 (29.6)	13.9 (36.2)
*I*/*σI*	10.2 (2.2)	19.0 (5.5)
CC_1/2_	0.995 (0.888)	0.988 (0.878)
***Refinement***		
Resolution range (Å)	35.66–1.23	46.0–1.24
Reflections used		
Working set/test set	84,196/4263	81,682/4027
*R*_work_	0.213	0.168
*R*_free_	0.240	0.200
Number of atoms	1689	1906
Protein	1503	1597
Water	186	309
Average B-factor (Å^2^)	22.3	14.0
Macromolecules	21.2	11.9
Solvent	31.1	24.8
Rmsd from ideality		
Bond length (Å)	0.006	0.018
Bond angles (˚)	0.917	1.636
Ramachandran plot		
Favored (%)	97.1	98.4
Outliers (%)	0	0

*Statistics for the highest-resolution shell are shown in parentheses

Superimposition of the SCM mutant structures was performed with PyMOL (https://pymol.org/2/). The coordinates of the SCM WT (PDB ID: 1MOL) and GFP-40 (PDB ID: 7CD7) [9], an SCM mutant containing the C41S mutation, were used for structural superimposition. For all mutants, the chain A of the two molecules in an asymmetric unit was used as a representative for superposition. Two loops between the β2 and β3 strands and between the β4 and β5 strands were ignored for the calculation of the C_α_ RMSD values since GFP-40 has mutations in these loops and the structural model of the SCM C41A mutant lacks these loops. Amino acid residues and water molecules within 4 Å of the 41st residue were listed using CONTACT in CCP4 package [29]. All figures illustrating the structures were generated using PyMOL.

## Results

### Design of SCM Mutants

To investigate the effect of mutation on protein stability, we designed SCM mutants in which the Cys41 residue was substituted with hydrophobic amino acids (Fig. 1b). We first designed a mutant substituting Cys41 with Val (namely C41V), whose side chain length is similar to that of Cys. Next, we aimed to investigate the effect of the volume of the side chain at the 41st residue on the stability of the protein. To this end, we designed two additional mutants in which Cys41 was substituted with Ala (C41A), a hydrophobic amino acid with a shorter side chain than Val, and Leu (C41L), a hydrophobic amino acid with a longer side chain than Val. In addition to the SCM C41A, C41V, and C41L mutants, the mutant in which Cys41 was substituted with a polar residue, Ser (C41S), was also prepared for comparison in this study. The SCM C41S mutant has been widely used in various studies [9–11, 17–21], and the crystal structures of the SCM mutants containing the C41S mutation have also been determined [9, 14]. Therefore, we comprehensively characterized five different SCM variants, SCM C41A, C41V, C41S, and wild type (WT) SCM.

### Characterization of the Purified SCM Mutants

**Fig. 2:**
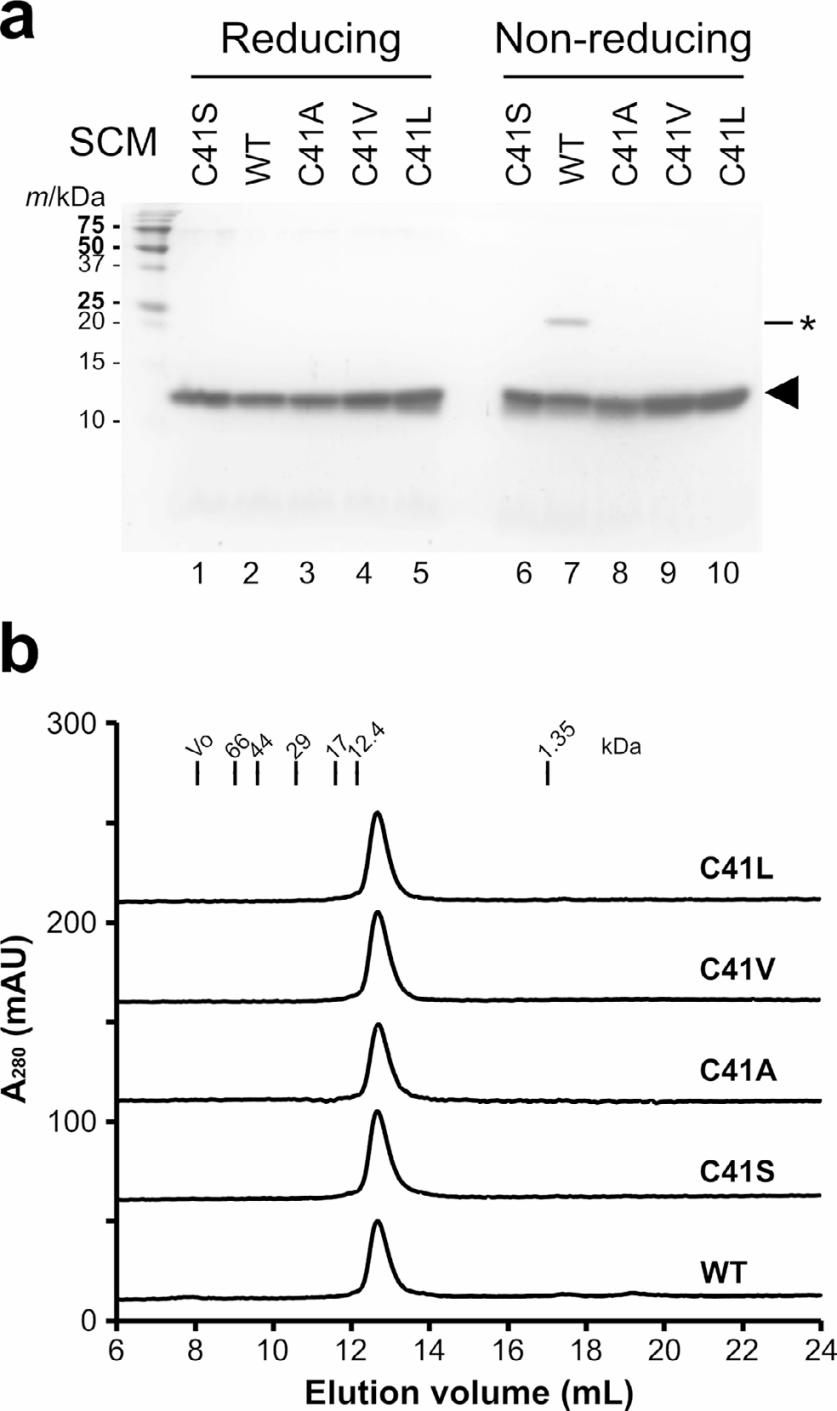
**Purification and size-exclusion chromatography (SEC) analyses of SCM mutants. a** SDS-PAGE analysis of purified SCM variants. 2 µg of protein was loaded on an 18% polyacrylamide gel. The band indicated by an asterisk is the covalent dimer of the WT SCM. **b** SEC profiles of purified SCM variants. Purified SCM variants were subjected to a size-exclusion chromatography column, equilibrated with 20 mM HEPES-Tris, 150 mM NaCl, pH 7.5. Chromatographs are shown with vertical offsets. The void volume (V_0_) and elution positions for bovine serum albumin (molecular mass: 67 kDa), ovalbumin (44 kDa), carbonic anhydrase (29 kDa), myoglobin (17 kDa), cytochrome *c* (12.4 kDa) and vitamin B_12_ (1.35 kDa) are indicated by vertical lines

Next, we expressed the SCM mutants in *E. coli* BL21(DE3) and purified the expressed proteins from the soluble fraction using Ni-NTA agarose. The N-terminal tag portion was then removed by treatment with TEV protease. All five SCM mutants were successfully purified (Fig. 2a). In general, all five SCM variants showed similar mobility under both reducing (lanes 1–5) and non-reducing (lanes 6–10) conditions. For the WT SCM protein, we detected an electrophoretic band that was likely the covalent homodimer under non-reducing condition (Fig. 2a, lane 7); this may be problematic for electrophoretic analyses.

The yields of the purified protein samples for the SCM mutants obtained from a 40 mL culture of *E. coli* were as follows: 1.6 mg for WT, 1.5 mg for C41S, 1.5 mg for C41A, 3.5 mg for C41V, and 2.0 mg for C41L. The yield of the C41V mutant was the highest among all the mutants, while the yields of C41A, C41L, and C41S were comparable to that of the WT, indicating no significant differences in expression levels among the SCM mutants.

Next, we analyzed the solution behavior of the SCM variants by size exclusion chromatography. All five SCM variants provided a single peak, indicating that they were monodisperse in solution (Fig. 2b). The apparent molecular masses estimated using the standard protein set (Table 2) were nearly identical to the calculated molecular mass (11.2 kDa), suggesting that at the concentration analyzed here the SCM variants behaved as monomers in solution.

**Table 2:** Apparent molecular mass of the SCM variants estimated by SEC

SCM variants	Apparent molecular mass (kDa)
WT	10.5
C41S	10.5
C41A	10.3
C41V	10.5
C41L	10.5

### Thermal Stability of SCM Mutants

**Fig. 3:**
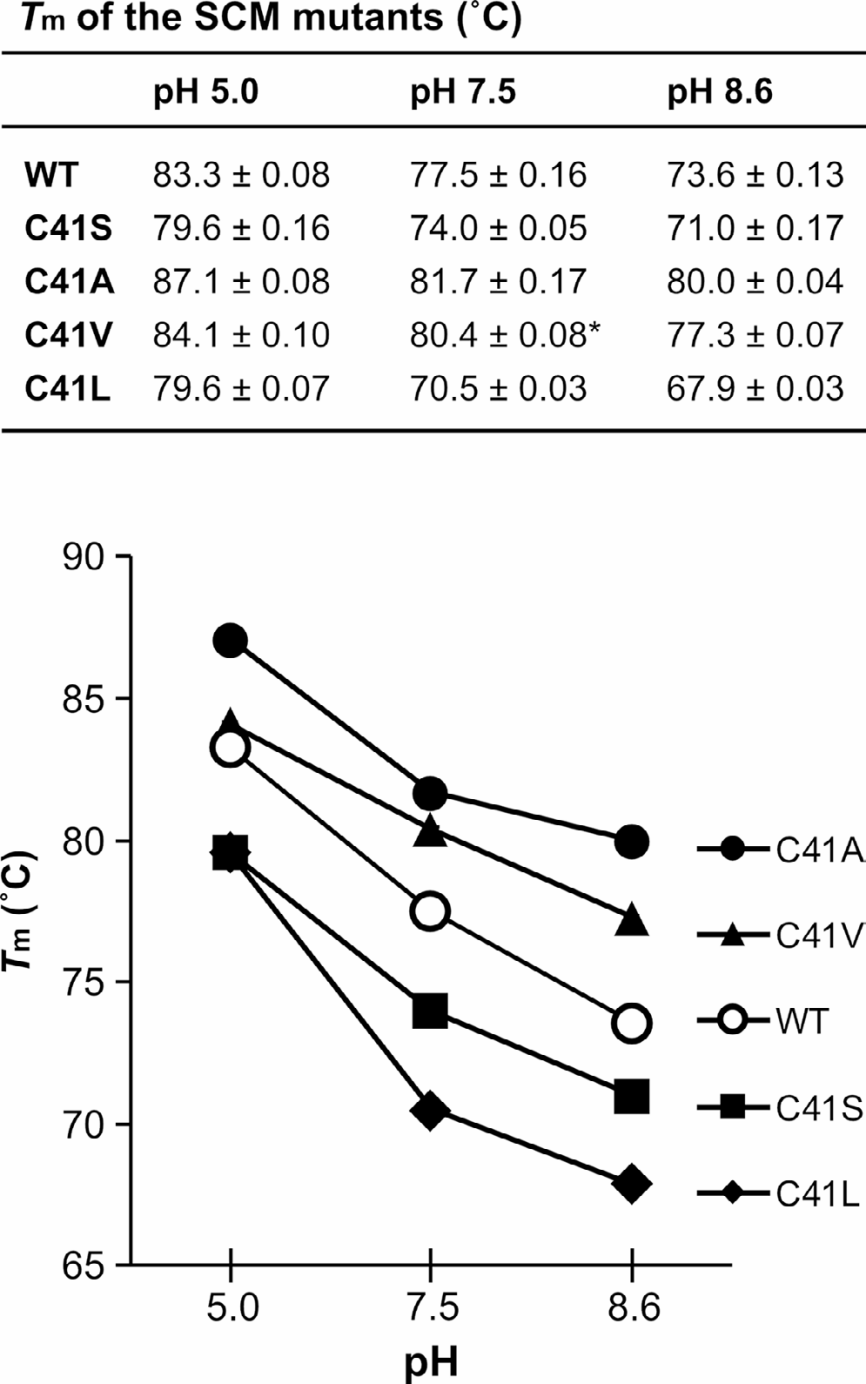
**Melting temperature (*T***_**m**_**) of the SCM variants at three different pH conditions.** The mean values of *T*_m_ (˚C) for each condition are shown together with the standard error (*n* = 4). Value marked with an asterisk (*) indicate data with *n* = 3

To investigate the thermal stability of the SCM mutants, we conducted differential scanning fluorimetry under three different pH conditions: acidic (pH 5.0), near-neutral (pH 7.5), and basic (pH 8.6). We chose pH 7.5 instead of pH 7.0 as near-neutral pH because it is close to the extracellular physiological pH commonly used for in vitro experiments. On the other hand, we chose pH 8.6 as the basic pH condition. This is because this pH is approximately one pH unit higher than pH 7.5, and the buffer solution used in this study has sufficient buffering capacity at this pH condition. Melting curves were obtained for all SCM mutants, thereby allowing the apparent thermal denaturation temperature (*T*_m_) values to be estimated for all three pH conditions (Fig. S1). The *T*_m_ at pH 7.5 of the SCM C41S mutant was estimated to be 74.0˚C (Fig. 3). This *T*_m_ value is similar to that obtained in our previous study (*T*_m_ = 74.2 °C) [9], indicating the methods for estimating *T*_m_ values employed in this study is superior in terms of reproducibility. For all SCM mutants, the *T*_m_ values were highest under acidic conditions and were progressively lower under neutral and basic conditions (Fig. 3). These results suggested that the SCM mutants were the most stable under acidic conditions (*T*_m_ at pH 5.0 = 87.1 °C, 84.1 °C, 83.3 °C, 79.6 °C, and 79.6 °C for C41A, C41V, WT, C41S, and C41L, respectively) and the most unstable under basic conditions (*T*_m_ at pH 8.6 = 80.0 °C, 77.3 °C, 73.6 °C, 71.0 °C, and 67.9 °C for C41A, C41V, WT, C41S, and C41L, respectively). Previous reports noted that the thermal stability of WT SCM was higher under acidic conditions than neutral conditions [3], and a similar result has been reported with respect to protein stability in the presence of denaturants [30]. The results obtained in this study were therefore consistent with those of previous studies. Furthermore, under all three different pH conditions the relative order of the *T*_m_ values of the SCM mutants was the same; that is, C41A > C41V > WT > C41S > C41L (Fig. 3). These results indicate that the substitution of Cys41 residue with Ser makes SCM unstable (Δ*T*_m_ of C41S at pH 5.0, pH 7.5, and pH 8.6 were − 3.7 °C, − 3.5 °C, and − 2.6 °C, respectively; Δ*T*_m_ = [*T*_m_ of mutant] – [*T*_m_ of wild type]), as expected, while substitution with Ala, or Val makes SCM slightly more stable (Δ*T*_m_ of C41A at pH 5.0, pH 7.5, and pH 8.6 were + 3.8 °C, + 4.2 °C, and + 6.4 °C, respectively, while Δ*T*_m_ of C41V at pH 5.0, pH 7.5, and pH 8.6 were + 0.8 °C, + 2.9 °C, and + 3.7 °C, respectively). It has been reported that substituting the Cys41 with Ala in another single-chain variant of monellin, MNEI, improves stability. The maximum heat resistance temperatures of the MNEI WT and its C41A mutant were 65 and 70 °C, respectively. The difference between them in the maximum heat resistance temperatures was 5 °C, indicating that substitution with Ala slightly affects the stability [31]. We also found that substitution of the Cys41 residue with Leu decreases the stability of SCM (Δ*T*_m_ of C41L at pH 5.0, pH 7.5, and pH 8.6 were − 3.7 °C, − 7.0 °C, and − 5.7 °C, respectively), indicating that the amino acid residues with side chains longer than Val make SCM proteins less stable.

### Determination of the Crystal Structures of the SCM C41A and C41V Mutants

Since SCM mutants in which Cys41 was substituted with Ala or Val residues exhibited slightly improved thermal stability compared to the WT, we next aimed to investigate the structural basis for this improvement. Therefore, we determined the crystal structures of the SCM C41A and C41V mutants and compared these structures to the structural information for the SCM WT and its mutant containing the C41S mutation, which are already available. The SCM C41A and C41V mutants were crystallized under slightly different conditions, resulting in having different space groups (i.e., *P*1 for C41A, and *P*2_1_ for C41V; Table 1). The crystals of both mutants contained two protein molecules that formed a dimer in the asymmetric unit (Fig. S2a). The mode of dimer formation in both SCM C41A and C41V was similar to that seen in the WT (Fig. S2b). Models for all 94 residues were successfully constructed for the C41V mutant, while the models for the loop between the β2 and β3 strands (i.e., residues Glu48–Arg51) in the C41A mutant could not be constructed due to poor electron densities. Therefore, these residues were ignored when calculating the root mean square deviation (RMSD) between the structures. In addition, the two molecules in an asymmetric unit were superimposed C_α_-RMSD values of 0.177 Å and 0.356 Å for SCM C41A and C41V, respectively. A similar comparison of two molecules in an asymmetric unit for the SCM WT (PDB ID: 1MOL) resulted in C_α_-RMSD value of 0.295 Å. Therefore, we considered the two molecules present in an asymmetric unit for these mutants to be equivalent with respect to structural information.

### Structural Comparisons of SCM Mutants

The overall structures of SCM C41A and C41V were almost identical to that of the WT. In fact, the structures of SCM C41A, C41V, C41S, and the WT were superimposed and found to overlap well in all combinations, and the main chain structures were almost identical except for the two flexible loops (Fig. S3). Furthermore, there is almost no difference in the main chain structure around the 41st residue and within the β2 strand where this residue is located (Fig. S3). This suggests that substituting C41 with Ala, Val, and Ser residues does not significantly affect the overall structure of the SCM protein.

**Fig. 4:**
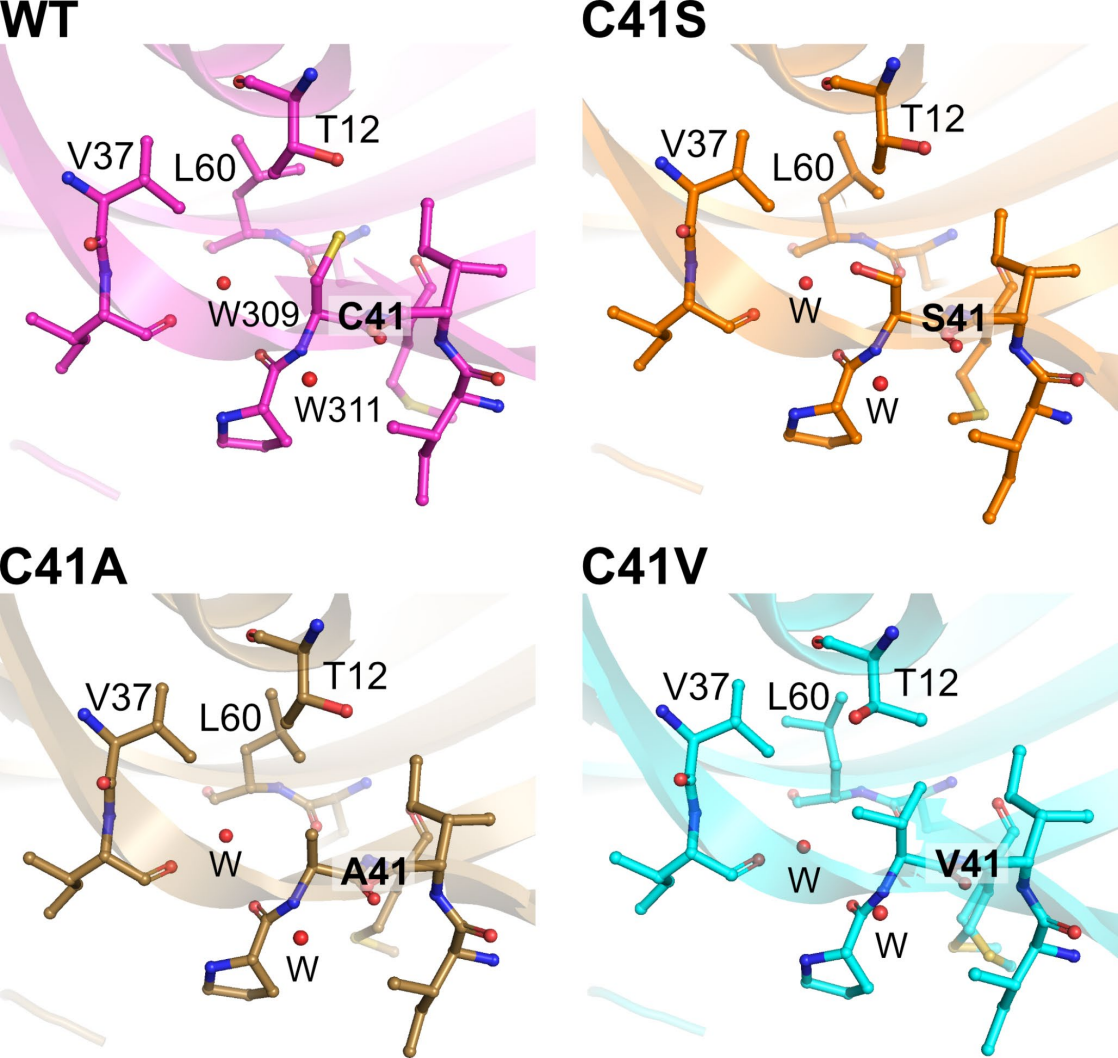
**Close-up view of the 41st residue of SCM variants.** Amino acid residues and water molecules within a 4 Å radius of the 41st residue are represented by stick models and red spheres, respectively. Two water molecules are conserved among the SCM variants, and their numbering is labeled in the WT structure (PDB ID: 1MOL)

**Fig. 5:**
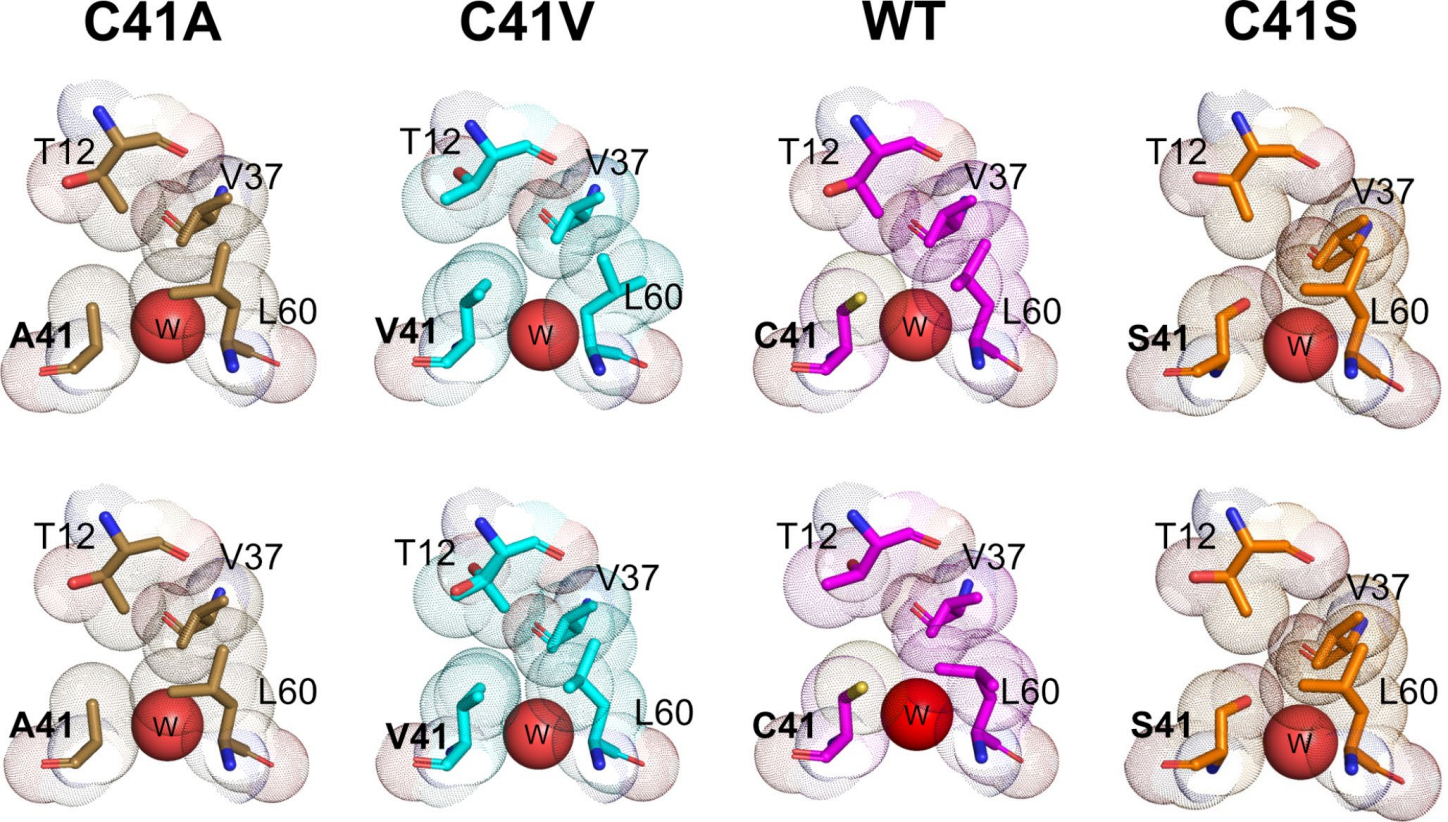
**Comparison of the conformations of amino acid residues around the 41st residue among SCM variants.** Shown are the structures around the 41st residue of both of the two molecules in the asymmetric unit for each SCM variant. The Thr12, Val38, and Leu60 residues, as well as the 41st residue, are represented as stick models, and the van der Waals radii of each of the atoms in these residues are depicted as dot models. Red spheres labeled with a “W” indicate oxygen atoms in the water molecules; this corresponds to W309 for the WT (PDB ID: 1 MOL). Thr12 in one of the molecules of the SCM C41V was modeled as a double conformer

Next, we focused on the orientations of the side chains of the amino acid residues present at and near the 41st residue. In all four SCM variants, Ile5, Ile6, Thr12, Val37, Ile38, Gln59, Leu60, Pro40, and Met42 residues were observed within 4 Å of the 41st residue, and two water molecules were also found in close proximity to this region (Fig. 4). The side chain of the 41st residue was oriented toward the hydrophobic region formed by Thr12, Val37, and Leu60, indicating that the polar side chain of Ser was not involved in the hydrophobic interactions that stabilize this structure. In fact, the methylene group of the side chain of the Ser41 residue did not make contact with Thr12, Val37, or Leu60. Instead, a hydroxyl group was observed to form a hydrogen bond with water inside the molecule in the SCM C41S mutant. A comparison of crystal structures revealed that the conformers of the side chains of Thr12 and Leu60 differed among the C41A, WT, and C41V variants (Fig. 5). Therefore, it appears highly likely that the orientations of the side chains of Thr12 on the α-helix and Leu60 on the β3-strand, which together comprise the hydrophobic region, are regulated by the properties of the 41st residue.

## Discussion

In this study, we demonstrated that substituting a single cysteine residue (Cys41) with small hydrophobic amino acids, such as Ala, and Val, can improve the thermal stability of the SCM protein (Fig. 3). The crystal structures of SCM mutants indicated that the side chains of Ala41 and Val41 are in close proximity to hydrophobic residues, as was the case for Cys41 (Fig. 4). The superior thermal stability observed after substitution with Ala or Val may therefore be attributed to the absence of their side-chain deprotonation, which can decrease thermal stability, as well as their interactions with surrounding residues. Indeed, it has been suggested that the deprotonation of the side chain of Cys residue is a factor that destabilizes single-chain variants of monellin in a pH-dependent manner [30, 32].

In the SCM C41A and C41V mutants, the main chain structures around the 41st residue were found to be almost identical to those of WT SCM and the C41S mutant (Fig. S3). Although no noticeable differences were found in the main chain structures, we observed differences in the orientations of the side chains of surrounding residues such as Thr12 and Leu60 among these variants (Fig. 5). In addition, it has been reported that double conformers were modeled at the Thr12, Leu62, and Cys41 residues in a high-resolution crystal structure of the single-chain monellin variant MNEI, although these residues were found in the hydrophobic core [7]. The high mobility of side chains of the residues in this hydrophobic core may allow for close contact with different sizes of side chains of hydrophobic residue. In this study, we discussed such conformational differences in peripheral side chains using only static structural information derived from X-ray crystallography. However, it is possible that differences in the conformational dynamics of the side chain may affect the thermal stability of the SCM protein. Furthermore, it is also important to test whether introducing mutations in the Cys41 residue affects the flexibility of the SCM backbone [33]. Using molecular dynamics simulations to generate information regarding the dynamics of molecules is required to provide a more detailed structural basis for the observed improvement in thermal stability.

The SCM C41A and C41V mutants have been useful in various studies because they eliminate the Cys residue without compromising thermal stability, as C41S does. However, we were unable to provide information on the folding process of either mutant in this study. Therefore, comparative studies of unfolding and refolding of the SCM C41A and C41V mutants relative to WT and C41S are necessary, as is the case with other model proteins [34] when these mutants are used as model proteins for protein folding studies.

In addition to physicochemical properties such as thermal stability, it is important to investigate whether the sweetness of the SCM mutants increases or decreases compared to the wild type. The crystal structures of the mutants containing the C41S, C41A, or C41V mutation showed that the overall structures were almost identical to that of the WT (Fig. S3) and the 41st residues were buried in the hydrophobic core of the protein (Fig. 4). These observations suggest that the mutations employed in this study do not significantly affect the interaction of the SCM mutants with the sweet taste receptor. However, the actual sweetness of the SCM mutants remains to be investigated in the future.

SCM serves as a molecular scaffold for antibody-like binder proteins, in which combinatorial length and amino acid residue mutations are introduced into two loops located between the β2 and β3 strands (loop 1) and the β4 and β5 strands (loop 2). Inserting a long amino acid sequence into loop 2 has been found to disrupt the steric structure [9]. As a result, the length of the loop that can be inserted into loop 2 is limited to 5 or 6 residues, which poses a challenge for generating SCM-based binder proteins [9]. Therefore, to allow for various loop sequences it is necessary to stabilize the SCM backbone. Since the Cys41 residue is far from these two loops and is not expected to directly affect the structure of either loop, introducing the C41A and C41V mutations may also be useful in improving the capacity of SCM to act as a molecular scaffold for binder proteins.

### Electronic Supplementary Material

Below is the link to the electronic supplementary material.

Supplementary Material 1

## Data Availability

The coordinates and structure factors for the SCM C41A and C41V have been deposited in the Protein Data Bank, under accession numbers 8JZ0 and 8JZ1. All data other than X-ray crystallography supporting the findings of this study are available within the paper and its Supplementary Information.

## References

[1] Morris JA, Cagan RH (1972). Purification of monellin, the sweet principle of Dioscoreophyllum cumminsii. Biochim Biophys Acta.

[2] Ogata C, Hatada M, Tomlinson G, Shin WC, Kim SH (1987). Crystal structure of the intensely sweet protein monellin. Nature.

[3] Kim SH, Kang CH, Kim R, Cho JM, Lee YB, Lee TK (1989). Redesigning a sweet protein: increased stability and renaturability. Protein Eng.

[4] Somoza JR, Jiang F, Tong L, Kang CH, Cho JM, Kim SH (1993). Two crystal structures of a potently sweet protein. Natural monellin at 2.75 a resolution and single-chain monellin at 1.7 a resolution. J Mol Biol.

[5] Tancredi T, Iijima H, Saviano G, Amodeo P, Temussi PA (1992). Structural determination of the active site of a sweet protein. A 1H NMR investigation of pMNEI. FEBS Lett.

[6] Spadaccini R, Crescenzi O, Tancredi T, De Casamassimi N, Saviano G, Scognamiglio R, Di Donato A, Temussi PA (2001). Solution structure of a sweet protein: NMR study of MNEI, a single chain monellin. J Mol Biol.

[7] Hobbs JR, Munger SD, Conn GL (2007). Monellin (MNEI) at 1.15 a resolution. Acta Crystallogr Sect F Struct Biol Cryst Commun.

[8] Farag MA, Rezk MM, Hamdi Elashal M, El-Araby M, Khalifa SAM, El-Seedi HR (2022). An updated multifaceted overview of sweet proteins and dipeptides as sugar substitutes; the chemistry, health benefits, gut interactions, and safety. Food Res Int.

[9] Yasui N, Nakamura K, Yamashita A (2021). A sweet protein monellin as a non-antibody scaffold for synthetic binding proteins. J Biochem.

[10] Konno T (2001). Multistep nucleus formation and a separate subunit contribution of the amyloidgenesis of heat-denatured monellin. Protein Sci.

[11] Szczepankiewicz O, Cabaleiro-Lago C, Tartaglia GG, Vendruscolo M, Hunter T, Hunter GJ, Nilsson H, Thulin E, Linse S (2011). Interactions in the native state of monellin, which play a protective role against aggregation. Mol Biosyst.

[12] Delfi M, Leone S, Emendato A, Ami D, Borriello M, Natalello A, Iannuzzi C, Picone D (2020). Understanding the self-assembly pathways of a single chain variant of monellin: a first step towards the design of sweet nanomaterials. Int J Biol Macromol.

[13] Sung YH, Shin J, Chang HJ, Cho JM, Lee W (2001) Solution structure, backbone dynamics, and stability of a double mutant single-chain monellin. structural origin of sweetness. J Biol Chem 276: 19624–19630. 10.1074/jbc.M100930200 10.1074/jbc.M100930200 11279156

[14] Leone S, Pica A, Merlino A, Sannino F, Temussi PA, Picone D (2016). Sweeter and stronger: enhancing sweetness and stability of the single chain monellin MNEI through molecular design. Sci Rep.

[15] Weiffert T, Linse S (2018). Protein stabilization with retained function of monellin using a split GFP system. Sci Rep.

[16] Delfi M, Emendato A, Leone S, Lampitella EA, Porcaro P, Cardinale G, Petraccone L, Picone D (2021) A super stable mutant of the plant protein monellin endowed with enhanced sweetness. Life (Basel) 10.3390/life11030236 10.3390/life11030236 PMC7999979 33809397

[17] Kimura T, Uzawa T, Ishimori K, Morishima I, Takahashi S, Konno T, Akiyama S, Fujisawa T (2005). Specific collapse followed by slow hydrogen-bond formation of beta-sheet in the folding of single-chain monellin. Proc Natl Acad Sci U S A.

[18] Xue WF, Szczepankiewicz O, Bauer MC, Thulin E, Linse S (2006). Intra- versus intermolecular interactions in monellin: contribution of surface charges to protein assembly. J Mol Biol.

[19] Xue WF, Szczepankiewicz O, Thulin E, Linse S, Carey J (2009). Role of protein surface charge in monellin sweetness. Biochim Biophys Acta.

[20] Assarsson A, Hellstrand E, Cabaleiro-Lago C, Linse S (2014). Charge dependent retardation of amyloid beta aggregation by hydrophilic proteins. ACS Chem Neurosci.

[21] Cabaleiro-Lago C, Szczepankiewicz O, Linse S (2012). The effect of nanoparticles on amyloid aggregation depends on the protein stability and intrinsic aggregation rate. Langmuir.

[22] Studier FW (2005). Protein production by auto-induction in high density shaking cultures. Protein Expr Purif.

[23] Hirata K, Yamashita K, Ueno G, Kawano Y, Hasegawa K, Kumasaka T, Yamamoto M (2019). ZOO: an automatic data-collection system for high-throughput structure analysis in protein microcrystallography. Acta Crystallogr D Struct Biol.

[24] Yamashita K, Hirata K, Yamamoto M (2018). KAMO: towards automated data processing for microcrystals. Acta Crystallogr D Struct Biol.

[25] Kabsch W (2010). XDS. Acta Crystallogr D Biol Crystallogr.

[26] McCoy AJ, Grosse-Kunstleve RW, Adams PD, Winn MD, Storoni LC, Read RJ (2007). Phaser crystallographic software. J Appl Crystallogr.

[27] Emsley P, Lohkamp B, Scott WG, Cowtan K (2010). Features and development of Coot. Acta Crystallogr D Biol Crystallogr.

[28] Liebschner D, Afonine PV, Baker ML, Bunkoczi G, Chen VB, Croll TI, Hintze B, Hung LW, Jain S, McCoy AJ, Moriarty NW, Oeffner RD, Poon BK, Prisant MG, Read RJ, Richardson JS, Richardson DC, Sammito MD, Sobolev OV, Stockwell, Adams PD (2019). Macromolecular structure determination using X-rays, neutrons and electrons: recent developments in Phenix. Acta Crystallogr D Struct Biol.

[29] Winn MD, Ballard CC, Cowtan KD, Dodson EJ, Emsley P, Evans PR, Keegan RM, Krissinel EB, Leslie AG, McCoy A, McNicholas SJ, Murshudov GN, Pannu NS, Potterton EA, Powell HR, Read RJ, Vagin A, Wilson KS (2011). Overview of the CCP4 suite and current developments. Acta Crystallogr D Biol Crystallogr.

[30] Aghera N, Dasgupta I, Udgaonkar JB (2012). A buried ionizable residue destabilizes the native state and the transition state in the folding of monellin. Biochemistry.

[31] Liu Q, Li L, Yang L, Liu T, Cai C, Liu B (2016) Modification of the Sweetness and Stability of Sweet-Tasting Protein Monellin by Gene Mutation and Protein Engineering. Biomed Res Int 2016:3647173 10.1155/2016/3647173 10.1155/2016/3647173 PMC4736911 26881217

[32] Maity H, Reddy G (2018). Thermodynamics and kinetics of single-chain Monellin folding with structural insights into specific collapse in the denatured state ensemble. J Mol Biol.

[33] Schilling J, Jost C, Ilie IM, Schnabl J, Buechi O, Eapen RS, Truffer R, Caflisch A, Forrer P (2022). Thermostable designed ankyrin repeat proteins (DARPins) as building blocks for innovative drugs. J Biol Chem.

[34] Yagi M, Sakurai K, Kalidas C, Batt CA, Goto Y (2003). Reversible unfolding of bovine beta-lactoglobulin mutants without a free thiol group. J Biol Chem.

