# Supplementary material for: Investigating the Effect of Substituting a Single Cysteine Residue on the Thermal Stability of an Engineered Sweet Protein, Single-Chain Monellin
Source: Protein J. 2023 Sep 22;42(6):698–708. doi: 10.1007/s10930-023-10154-0 (PMC10590300; doi:10.1007/s10930-023-10154-0)
Supplement: Supplementary file 1 — Supplementary Material 1 [file 10930_2023_10154_MOESM1_ESM.pdf]

Supplementary Information for:

Investigating the Effect of Substituting a Single Cysteine Residue on the Thermal Stability of an Engineered Sweet Protein, Single-Chain Monellin

Kyosuke Ohnuma[1], Atsuko Yamashita[2], Norihisa Yasui[2]

[1]School of Pharmaceutical Sciences, Okayama University, Okayama, Japan. [2]Graduate School of Medicine, Dentistry and Pharmaceutical Sciences, Okayama University, Japan

Corresponding Author

Norihisa Yasui
E-mail: nyasui@okayama-u.ac.jp
ORCID: 0000-0001-7117-3070

Table S1. The sequences of the primers used for mutagenesis.

Name	Sequence
SCM_S41C_F	CCGTCCGtGCATGAAAAAAACCATCTATGAAAAC
SCM_S41C_R	TTCATGCaCGGACGGATCACTTTGTTAAAGGTC
SCM_S41A_F	CCGTCCGgcCATGAAAAAAACCATCTATGAAAAC
SCM_S41A_R	TTCATGgcCGGACGGATCACTTTGTTAAAGGTC
SCM_S41V_F	CCGTCCGgtCATGAAAAAAACCATCTATGAAAAC
SCM_S41V_R	TTCATGacCGGACGGATCACTTTGTTAAAGGTC
SCM_S41L_F	CCGTCCGctgATGAAAAAAACCATCTATGAAAAC
SCM_S41L_R	TTTCATcagCGGACGGATCACTTTGTTAAAGGTC

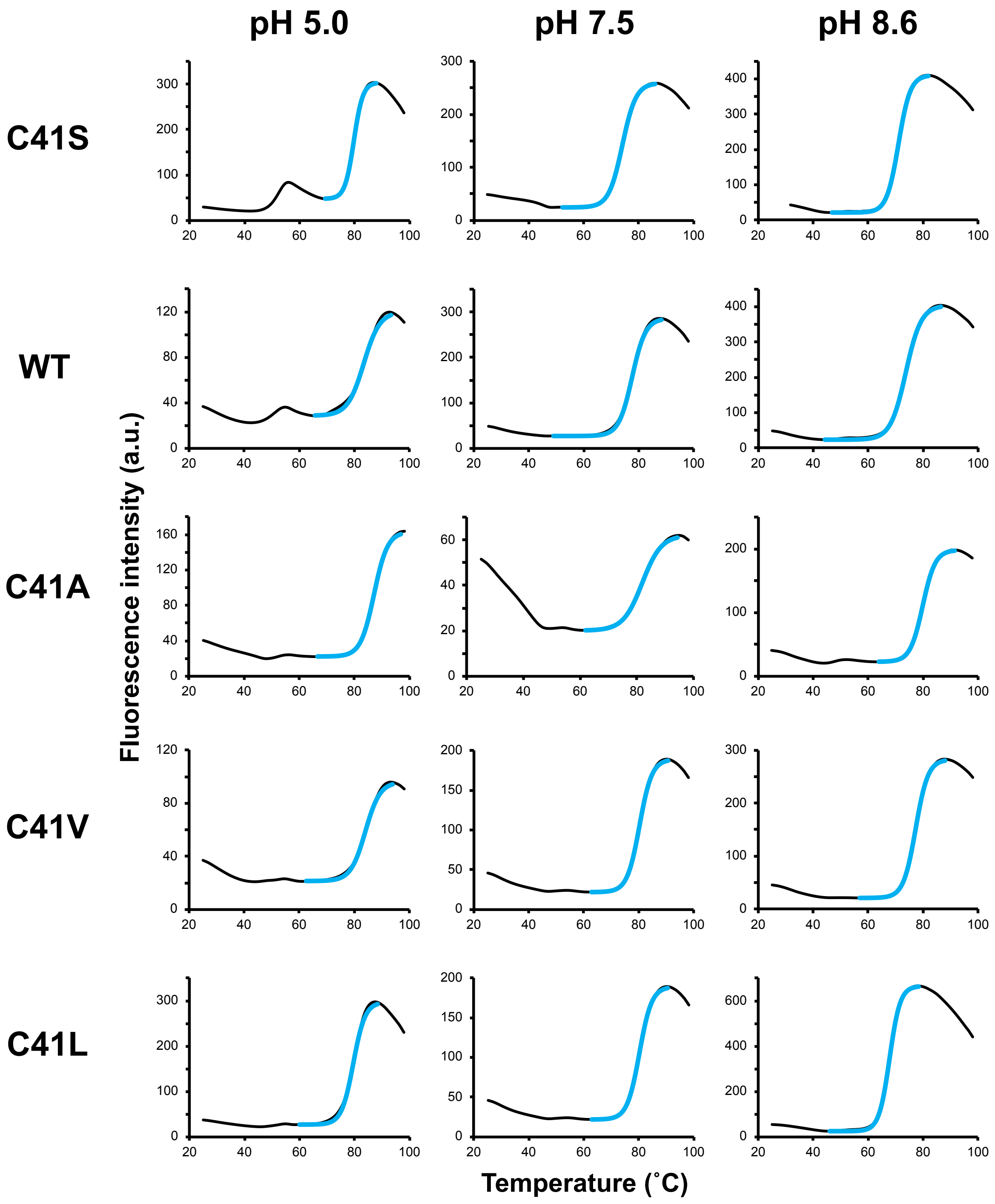

Figure S1. Differential scanning fluorimetry analysis of the thermal stability of SCM mutants. The representative thermal melt curves of each measurement and the fitting curves for a two-state Boltzmann model are shown in black and cyan, respectively

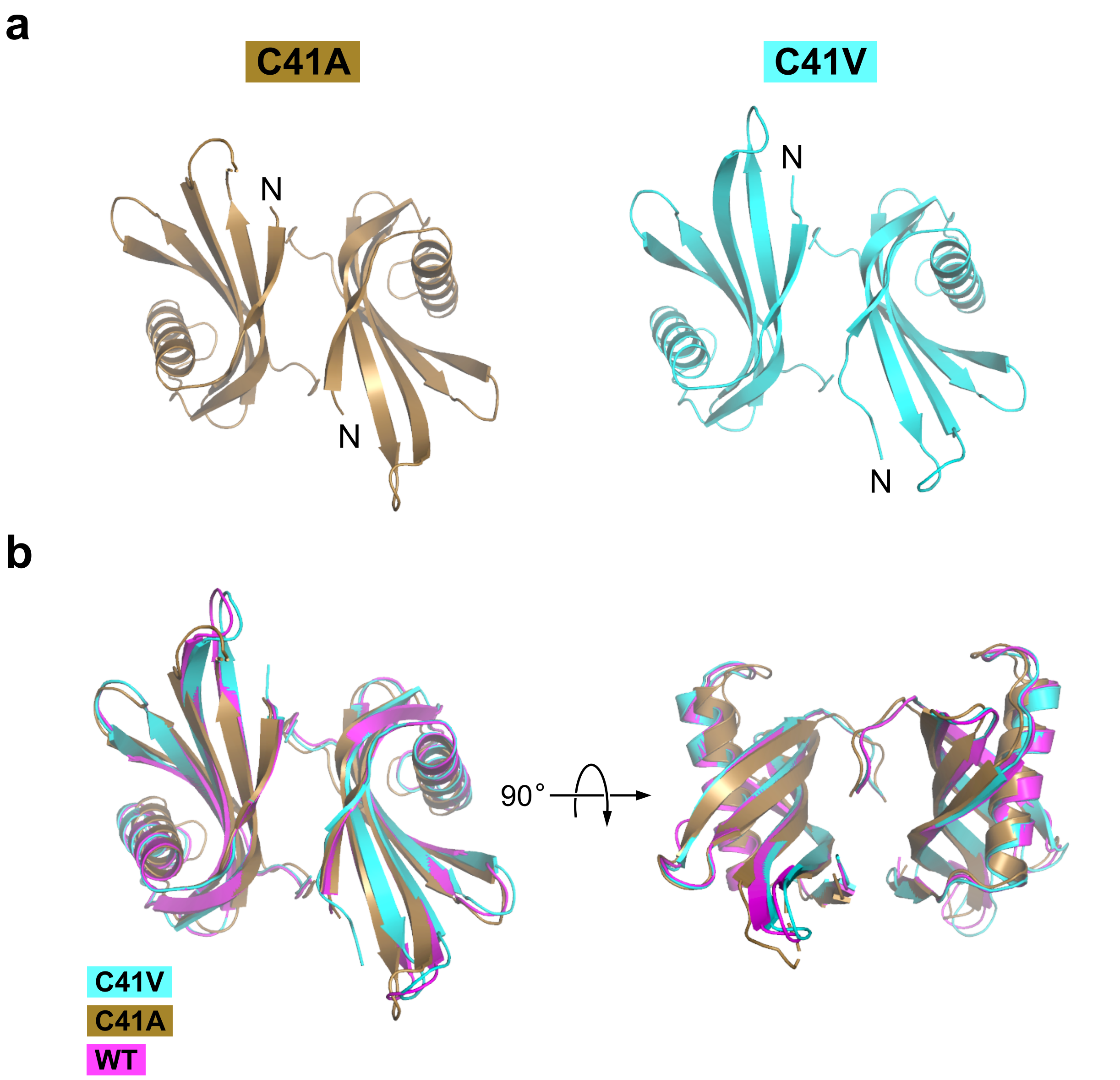

Figure S2. Structure of the SCM C41A and C41V mutants. a Overall structures of the SCM C41A and C41V mutants. Two molecules in an asymmetric unit are shown. The N-terminus of each molecule was labeled. **b** Superposition of the dimer on the asymmetric units of SCM C41V, C41A, and the WT SCM

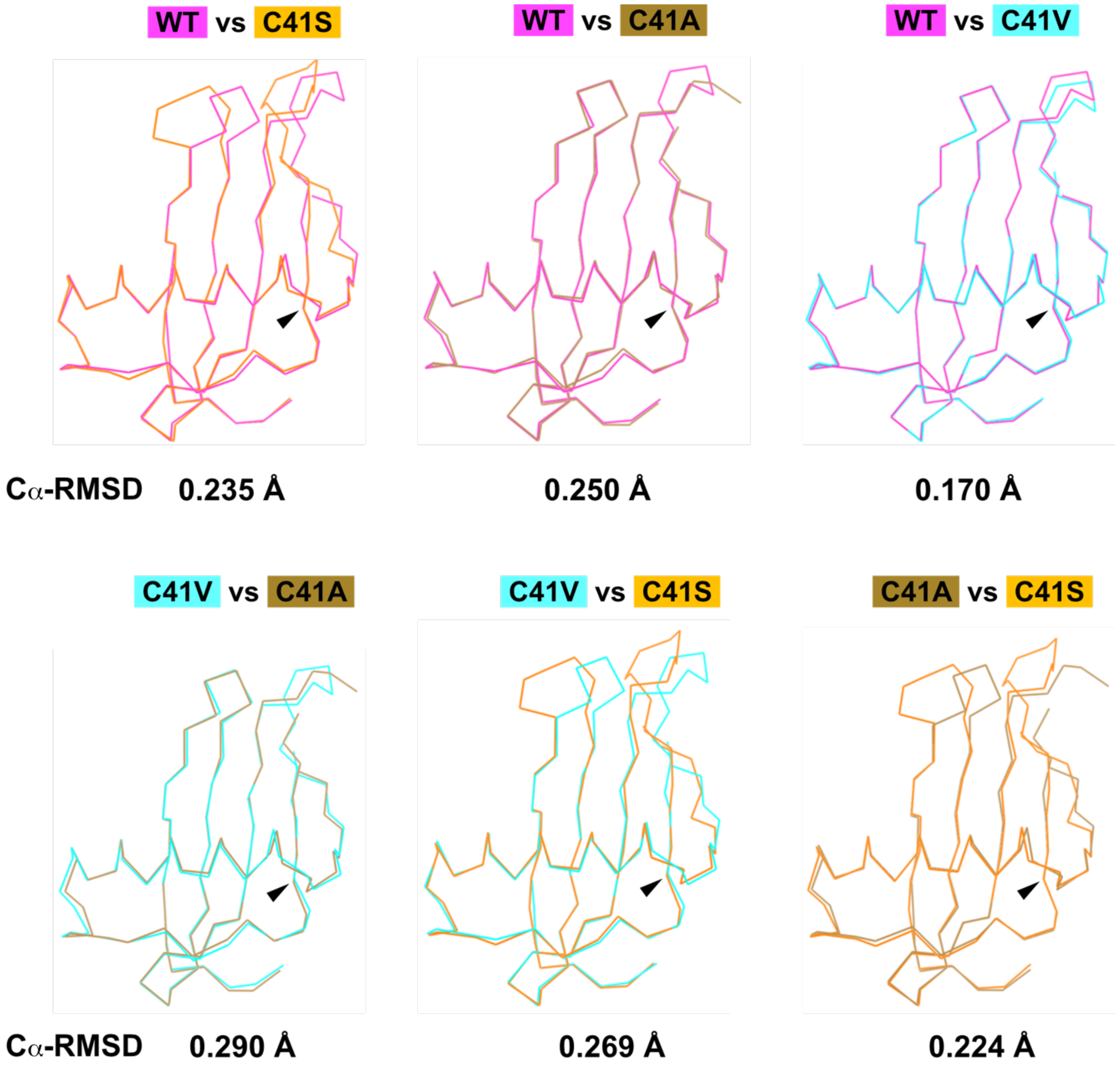

Figure S3. Structures of SCM C41A and C41V. Superpositions of main chain structures of the SCM mutants (i.e., WT, C41S, C41A, and C41V), with C_α-RMSD indicated under each superposition. Two loops between the β2 and β3 strands and between the β4 and β5 strands were ignored for C_α-RMSD calculations. The position of the 41st residue is indicated by an arrowhead
